# Supplementary material for: TRPV1 channels are critical brain inflammation detectors and neuropathic pain biomarkers in mice
Source: Nat Commun. 2017 May 10;8:15292. doi: 10.1038/ncomms15292 (PMC5436240; doi:10.1038/ncomms15292)
Supplement: Supplementary Information — Supplementary Figures and Supplementary References. [file ncomms15292-s1.pdf]

<u>**Supplementary Figures**</u>

Supplementary Figure 1: TRPV1 expression in the ACC of both naïve and CCI young mice.

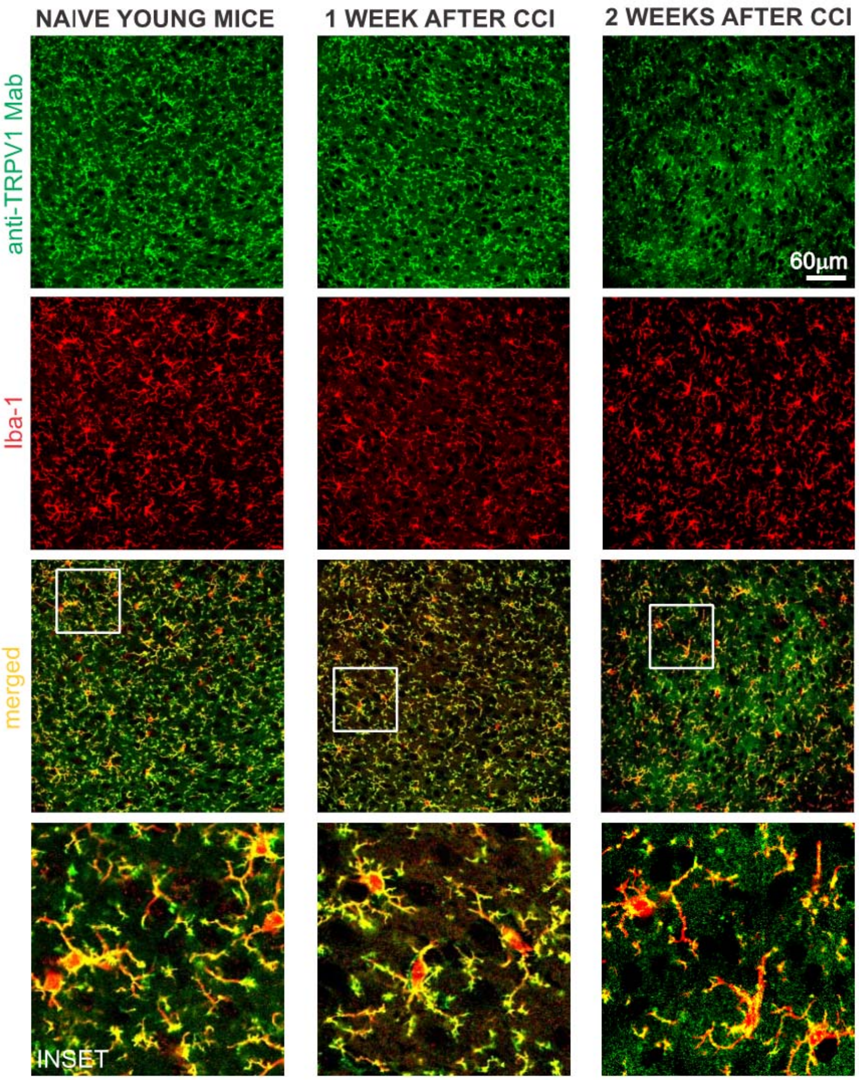

As in adult mice, the anti-TRPV1 MAb (green label) stains cortical microglial processes and somas (red label) of naïve young mice, as shown by the colocalization (in yellow) between the anti-TRPV1 and the iba-1.
Unlike CCI adult animals, TRPV1 is purely expressed in the microglia of the ACC of 1-week CCI young mice (4 experiments from 4 mice, n=4/4). Considering the faster cellular reproduction in youngs, TRPV1 expression has been tested in the ACC sections from 2-weeks CCI young mice. Also at this time point, TRPV1 is selectively expressed in microglia cells (n=2/2). Same result is observed in the ACC of 3 days CCI young mice (data not shown).

Supplementary Figure 2: TRPV1 antibody validation.

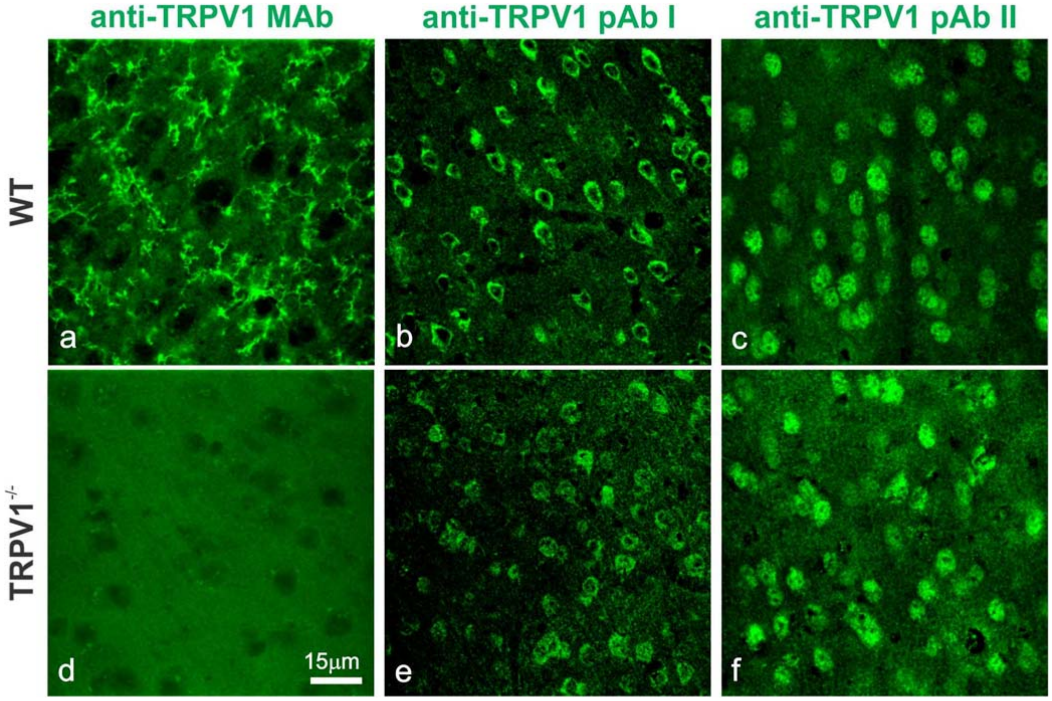

High resolution confocal laser scanning photomicrographs of ACC sections **from the same wt (a-c) and the same *TRPV1$^{-/-}$* mice (d-f),** showing the staining pattern of three different anti-TRPV1 antibodies (Abs). (**a**) Anti-TRPV1 MAb labeling of fibers (Millipore Chemicon, 1:100). Same protocol of immunostaining on *TRPV1$^{-/-}$* mice tissues (**d**) shows no stain from both primary or secondary antibody (the latter tested on the background controls of secondary Ab2s; data not shown). (**b,c**) The cell cytoplasm or whole-cell body labelling of two anti-TRPV1 pAbs is similar in cortical tissues from wt and -/- mice (**e,** Immunological Sciences, 1:100**; f.** Neuromics, 1:500). Scale bar, 15 µm.

Supplementary Figure 3: Both Iba-1 and TRPV1 signals were higher in young than adult mice.

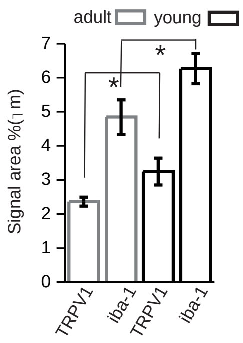

Quantification of the area filled by the TRPV1 signal and Iba-1 one. In cortical sections from naïve young mice both TRPV1 (n=12/8) and Iba-1 (7/6) signal areas are

significantly larger compare adult animals (n=16/9 for TRPV1, n=6/6 for Iba-1; *$p<0.05$).

Supplementary Figure 4: Expression of TRPV1 mRNA and protein in microglial cells, neurons and mouse tissue

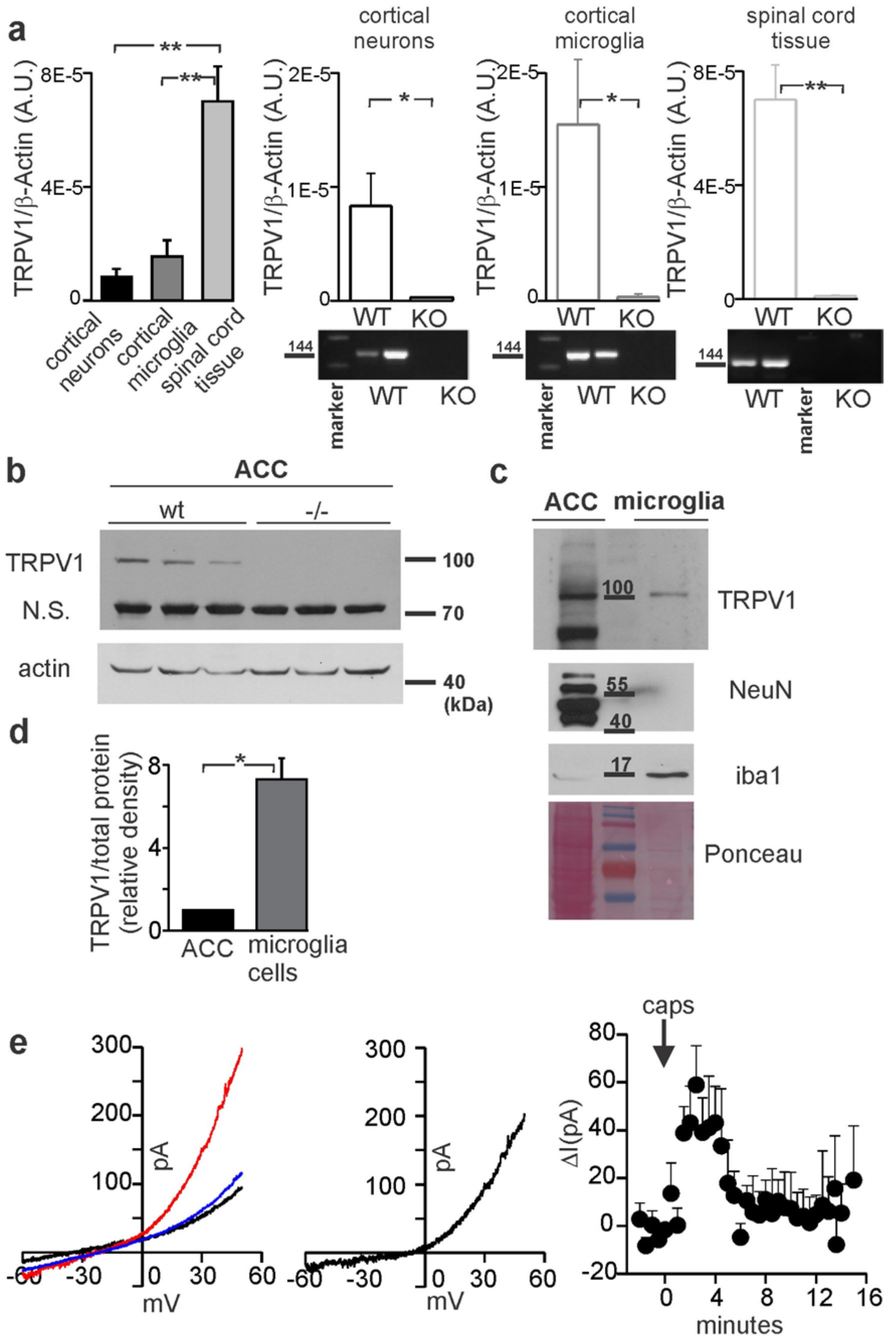

(**a**) RT-PCR of TRPV1 gene expression in cortical neurons, microglial cells and spinal cord isolated from C57BL6J or *TRPV1*$^{-/-}$ mice: for each sample, TRPV1 threshold cycle was normalized to that of β-actin. Data are expressed as mean±SD and are representative of four independent pools of mouse cortices (3-4 mice/pool) (**$p<0.01$ one-way ANOVA for left panel and *$p<0.05$ and **$p<0.001$ Student t-test

for the remnant panels). A representative agarose gel (2%) electrophoresis of two samples per cell type/tissue is also shown in order to display the specificity of RT-PCR reactions. (**b**) Representative immunoblot of TRPV1 expression in ACC from wild-type (wt) and *TRPV1*$^{-/-}$ mice. Protein lysate of each tissue was subjected to immunoblotting following SDS-10% PAGE against the anti-TRPV1 monoclonal antibody. The bottom portion of the nitrocellulose membranes was probed with the anti–actin antibody, as loading control. N.S.: non specific band. Note that this band is still present in tissue from *TRPV1*$^{-/-}$ mice. (**c**) Representative immunoblots comparing the TRPV1 content in ACC (50 μg) and microglial cells (6 μg). Microglial cells isolated from mice were lysed and subjected to immunoblotting against the anti-TRPV1 antibody. Note that N.S. band is absent in microglia cell cultures. Due to a strong difference in relative abundance of actin in the protein extracts of ACC tissues and isolated microglial cells (data not shown), the enrichment in TRPV1 expression was assessed by normalizing optical density values for the Ponceau staining of total proteins[74]. Particularly, optical density values were internally normalized by Ponceau staining of total protein content and further corrected for the value of ACC considered equal to 1. *$p<0.05$ *vs* ACC. The purity of microglial cultures was assessed by assessing specific markers for neurons (NeuN) and microglia cells (Iba1). (**d**) Bar graph of summary data of normalized densitometric ratios ± S.D. of three separate experiments. (**e**) *Left*, Current-voltage relationship of the capsaicin-induced current by application of capsaicin 1μM in a microglial cell from acute cortical slice of *CX3CR1*$^{+/GFP}$ mouse before (black curve), during (red curve) and after (blue curve) capsaicin application; *Middle*, capsaicin induces an outward rectifying current with reversal potential at about 0 mV (results obtained by subtracting the current before and after the capsaicin application). *Righ*t, time plot of the mean current amplitude at +50 mV (n=9). The arrow indicate 3-9 minutes of capsaicin application.

Supplementary Figure 5: TRPV1 distribution in astrocytes of the ACC and hippocampus of adult naïve mice.

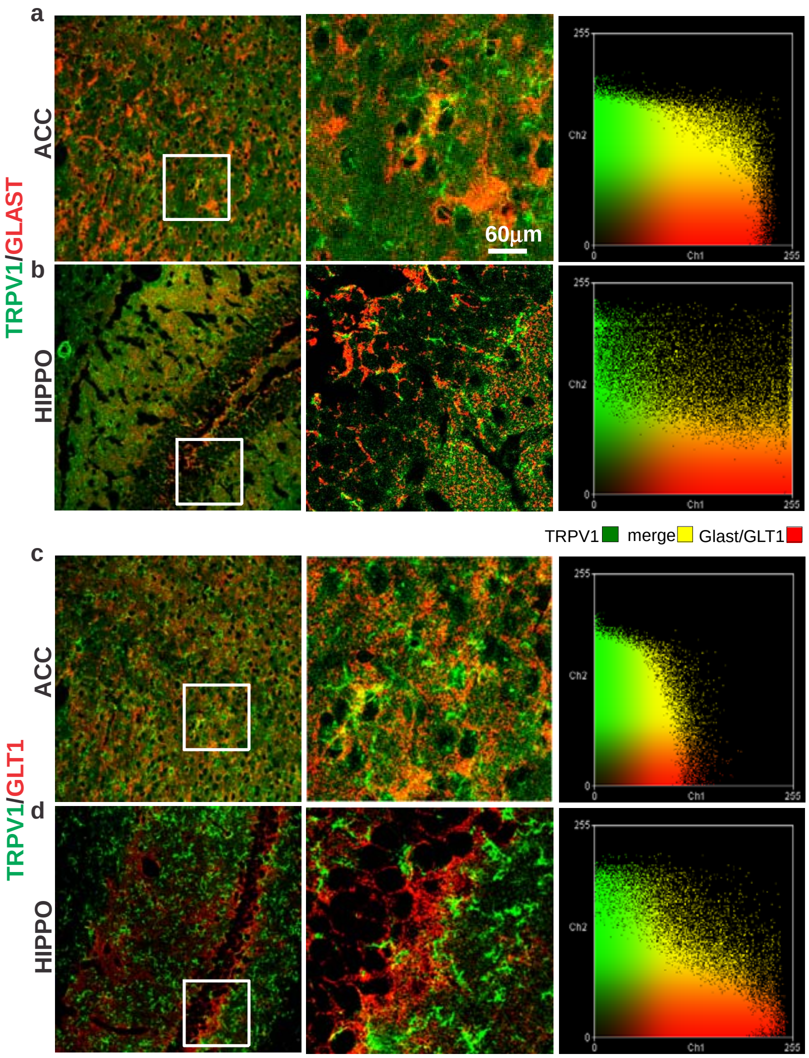

(**a,d**) Representative photographs of anti-TRPV1 and anti-GLAST (**a,b**) or anti-GLT1 (**c,d**) positive astrocytes of ACC (**a,c**) and hippocampus (HIPPO; **b,d**). Merging areas magnifications are presented in the middle panels. Scale bar, 60µm**.** In the right panels, correlative color scatter plots of TRPV1 (green) and GLAST/ GLT1 (red) for the single experiment reported in a,b,c,and d, show the colocalization (yellow) of the two antibodies (in **a,** r= 0.046; in **b,** r= 0.17; in **c,** r= 0.043; in **d** r= 0.194).

Supplementary Figure 6: TRPV1 is distributed in the microglia of other brain areas.

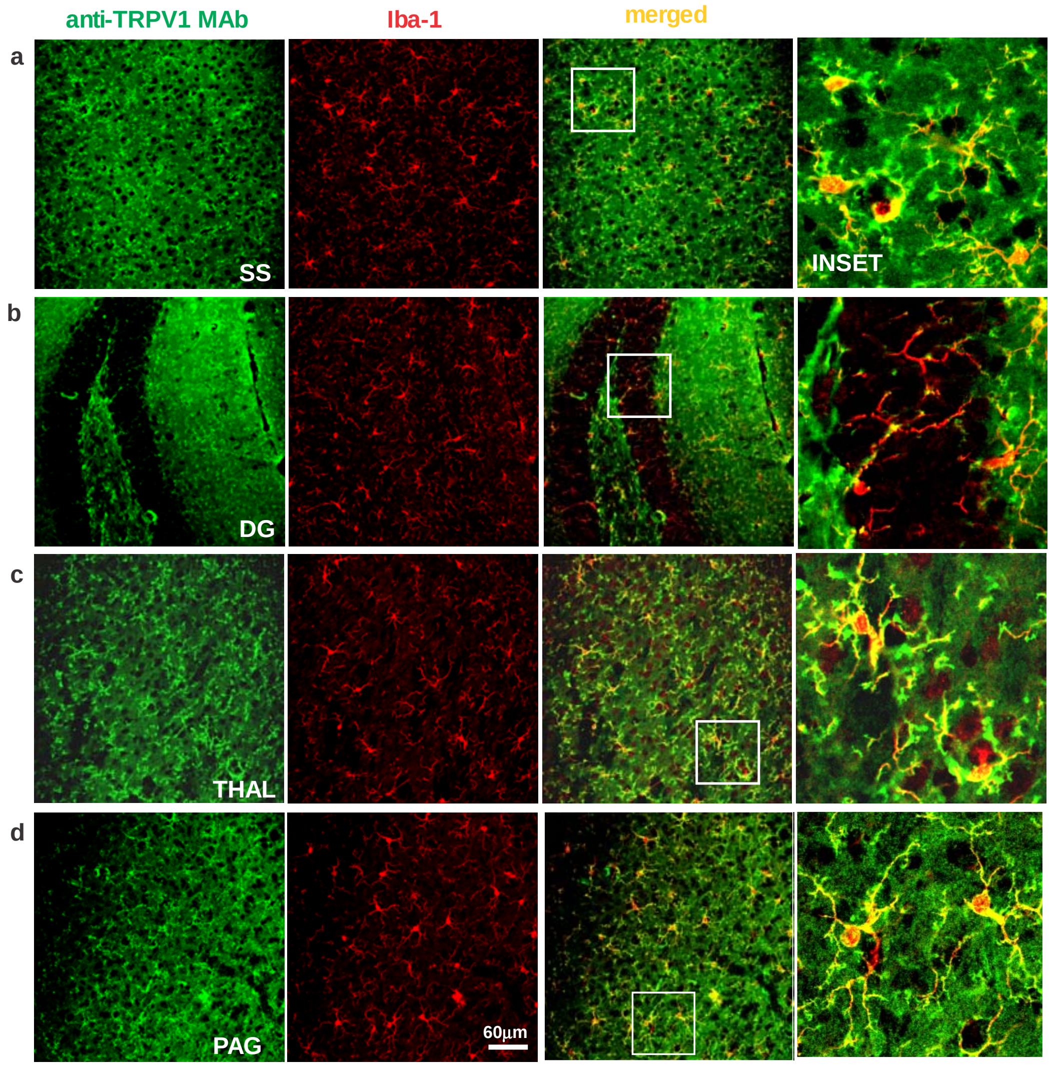

(**a-d**) Confocal laser scanning photomicrographs of somatosensory cortex (SS n=17/9), dentate gyrus (DG n=17/9), thalamus (THAL n=17/9) and periaqueductal gray (PAG n=11/6) showing labeling for anti-TRPV1, Iba-1 and their merged. Like the ACC, also in these brain regions the TRPV1 is mainly expressed in the microglia. Scale bar, 60μm for the lower magnification images.

Supplementary Figure 7: In the dorsal horn of naïve mice TRPV1 is also expressed in neurons.

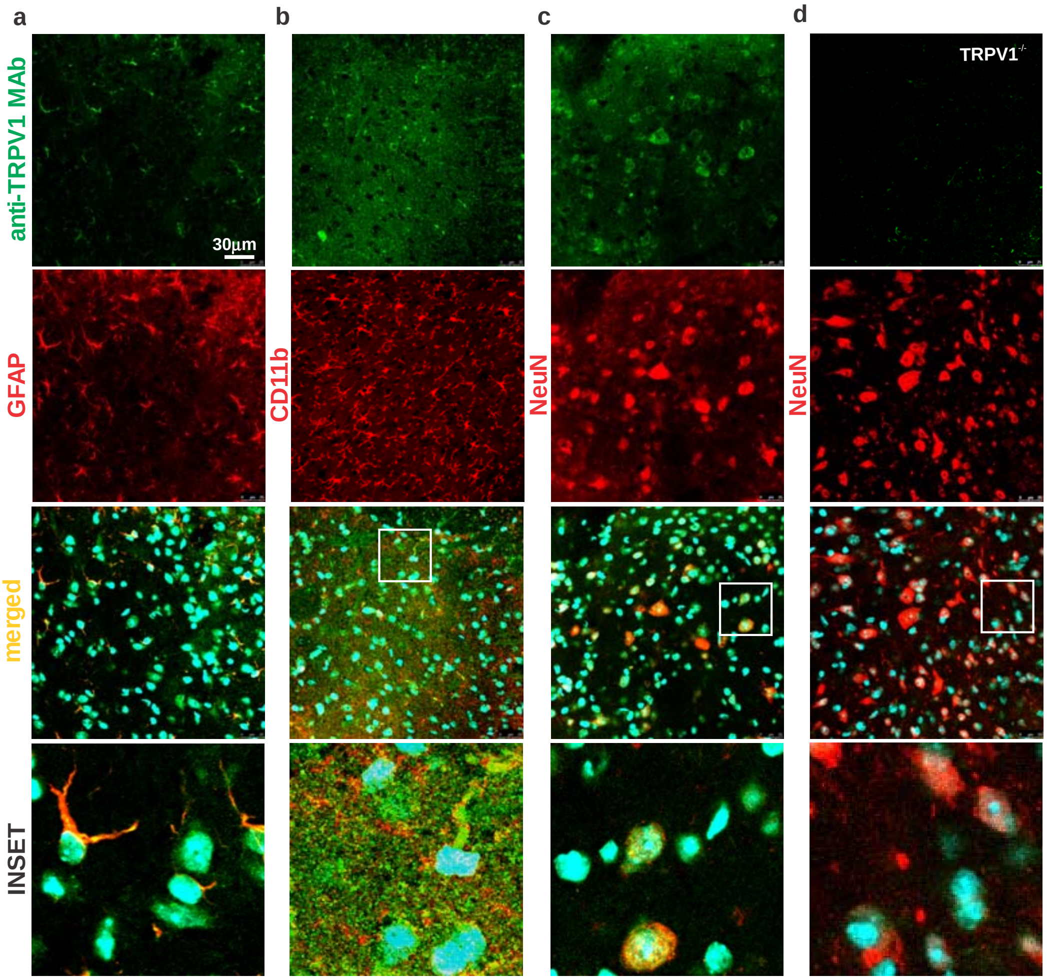

Representative confocal images of spinal cord dorsal horn sections immunolabeled with the TRPV1 Mab (green), anti- GFAP, CD11b, and NeuN (all red) from WT (**a,b,c**) and *TRPV1-/-* mice (**d**). Differently from brain areas, TRPV1 is also present in neurons of spinal cord naïve mice (n=4/4). Scale bar, 30µm for the lower magnification images.

Supplementary Figure 8: TRPV1 is expressed in both glutamatergic and GABAergic neurons of the dorsal and ventral horn

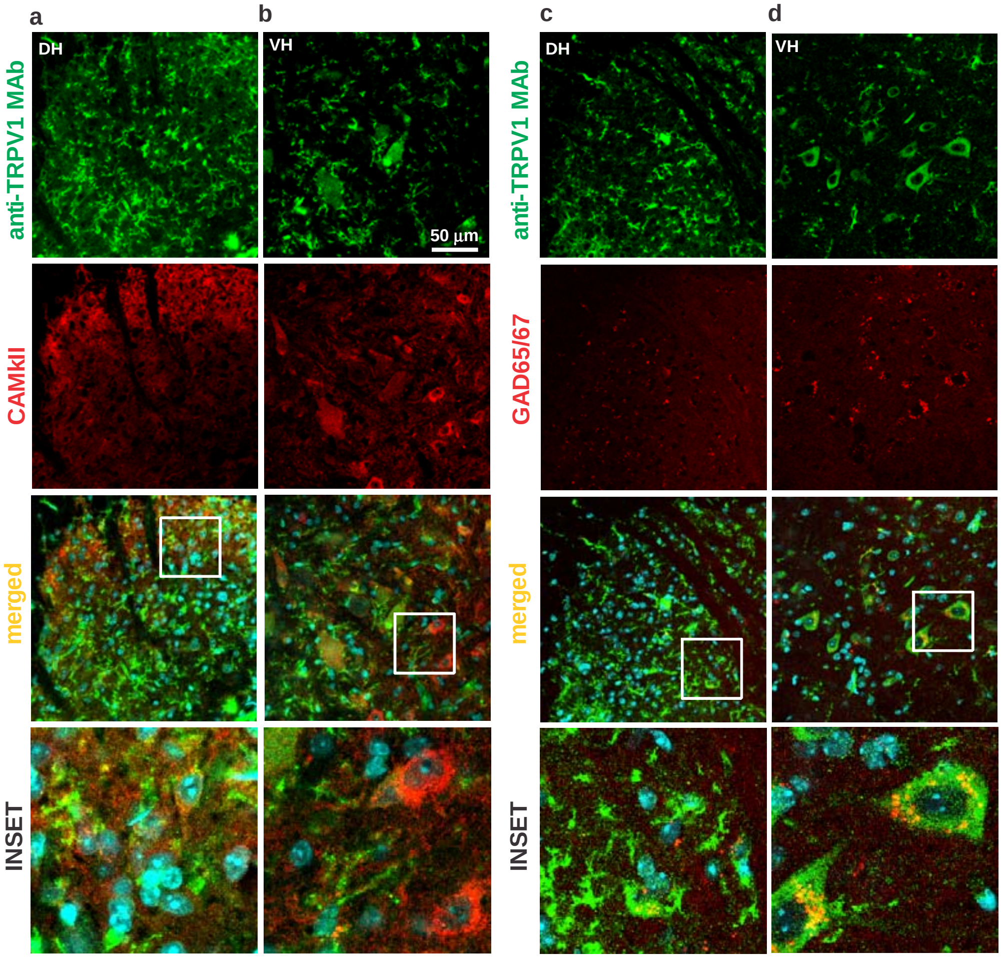

(**a,b**)Photomicrographs of immunofluorescence for the anti-TRPV1 MAb (green) and anti-CAMKII (red) in dorsal (DH) and ventral horn (VH). CAMKII is highly and diffusely expressed in DH and less in VH of naïve animal spinal cord. The anti-TRPV1 MAb is highly expressed and partially co-localizes with CAMKII present in body cells of both horns (merged panels-yellow). (**b,c**) Photomicrographs of double immunofluorescence for anti-GAD65/67 (red) and anti-TRPV1 MAb (green). The GAD65/67 is stained as dot-like pattern in DH and VH of naïve animal spinal cord. The anti-TRPV1 MAb is highly expressed and partially co-localizes with the anti-GAD65/67 present in body cells (merged panels - yellow).

Note that the TRPV1 expression on VH neurons may provide a preliminary evidence on a possible involvement of TRPV1 signaling in the nocifensive behavior.

Supplementary Figure 9: anti-TRPV1 MAb staining pattern in adult WT and *TRPV1*$^{-/-}$ DRG neurons

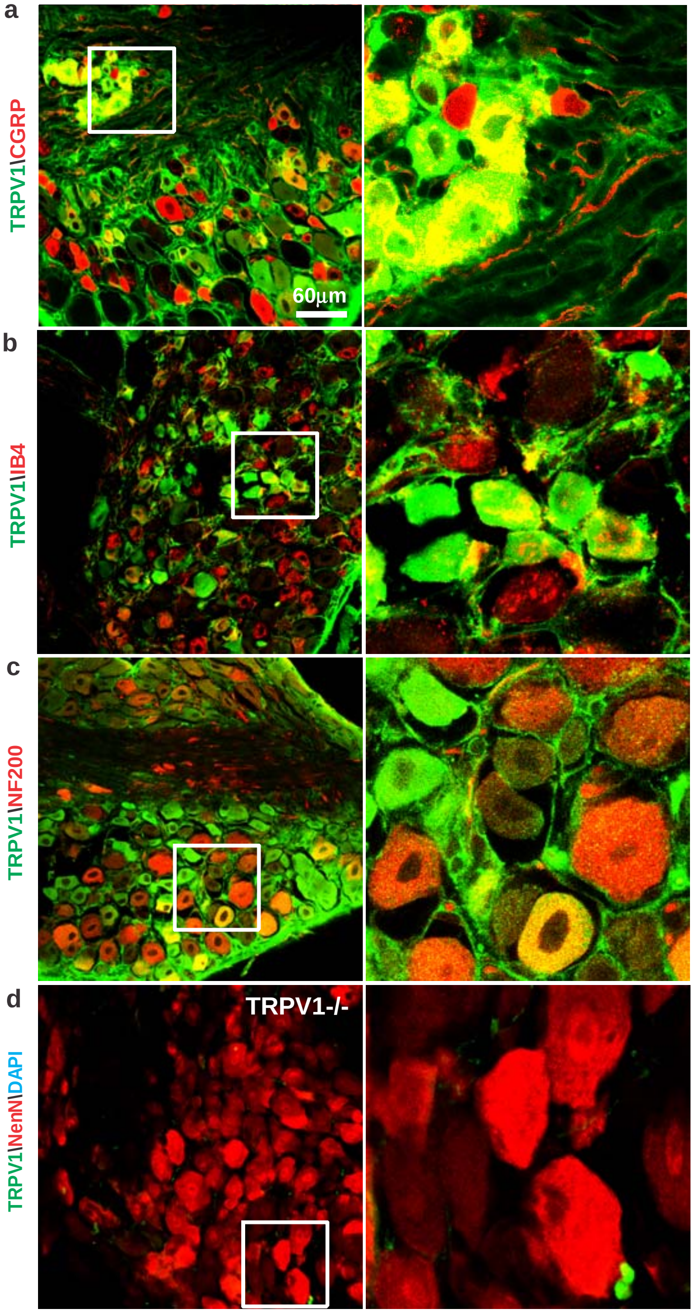

40x confocal images (left panels) and detail magnification (right panels) of DRG sections. Double staining of anti-TRPV1 MAb (green) with anti-CGRP (**a**), anti-IB4 (**b**) anti-NF200 (**c**, all in red) in DRG neurons of WT animals (n=4) indicates that part of neurons identified into each subgroup expressed TRPV1 (yellow; 32% of CGRP^{+} neurons are TRPV1+, 14.5 % of IB4+ neurons are TRPV1+, 14 % NF100+ cells were TRPV1+). (**d**) In *TRPV1*$^{-/-}$ DRG no cells show labelling for TRPV1 MAb (n=3). Scale bar, 60µm for the lower magnification images.
Note that the NF200+ neurons might correspond to the heat-sensitive myelinated nociceptors[90].

Supplementary Figure 10: TRPV1 distribution pattern in 3 days CCI adult mice.

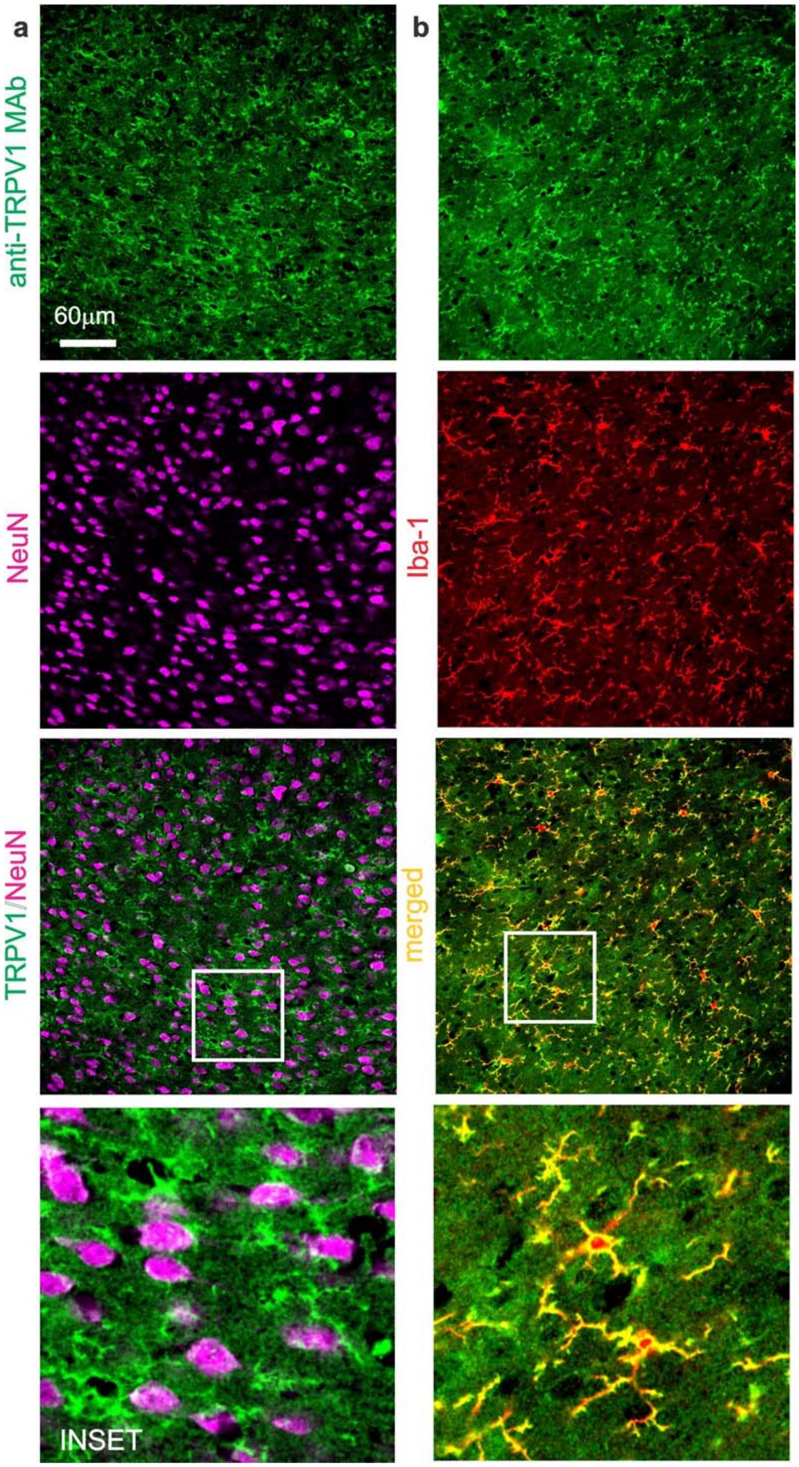

Double immunostaining for (**a**) TRPV1 (in green) and neurons (purple label), and for (**b**) TRPV1 and microglial cells (in red) from ACC sections of 3 days CCI mice. Anti-TRPV1 positive fibers surround neuronal bodies and are fully labeled with the iba-1(merging points in yellow). The TRPV1 expression pattern in the cortex of 3 days CCI mice were similar to naïve mice (see Fig 1). Scale bar, 60μm for the lower magnification images.

Supplementary Figure 11: Anti-TRPV1 neuronal labeling was absent in CCI *TRPV1*$^{-/-}$ mice.

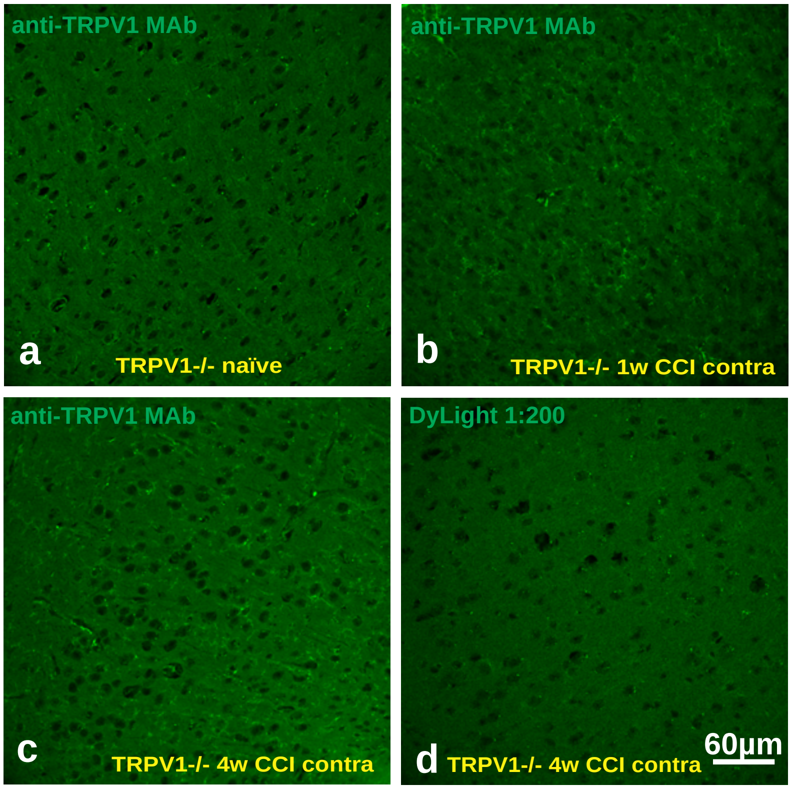

Confocal laser scanning photomicrographs of ACC sections containing superficial layers from naïve (**a**), 1 week (**b**) and 1 month CCI TRPV1$^{-/-}$ mice (**c,d**) stained with the anti-TRPV1 MAb (Millipore Chemicon, 1:100) (**a-c**) or background control of secondary Ab (**d**). Anti-TRPV1 MAb shows no staining in naïve condition (**a**, n=4) and neither at 1 week (**b**, n=3) nor at 4 weeks (**c**, n=6) after CCI. Background control of secondary Ab shows no false staining (**d**).

Supplementary Figure 12: Capsaicin-induced increase of mEPSC is due to AMPA receptors activation

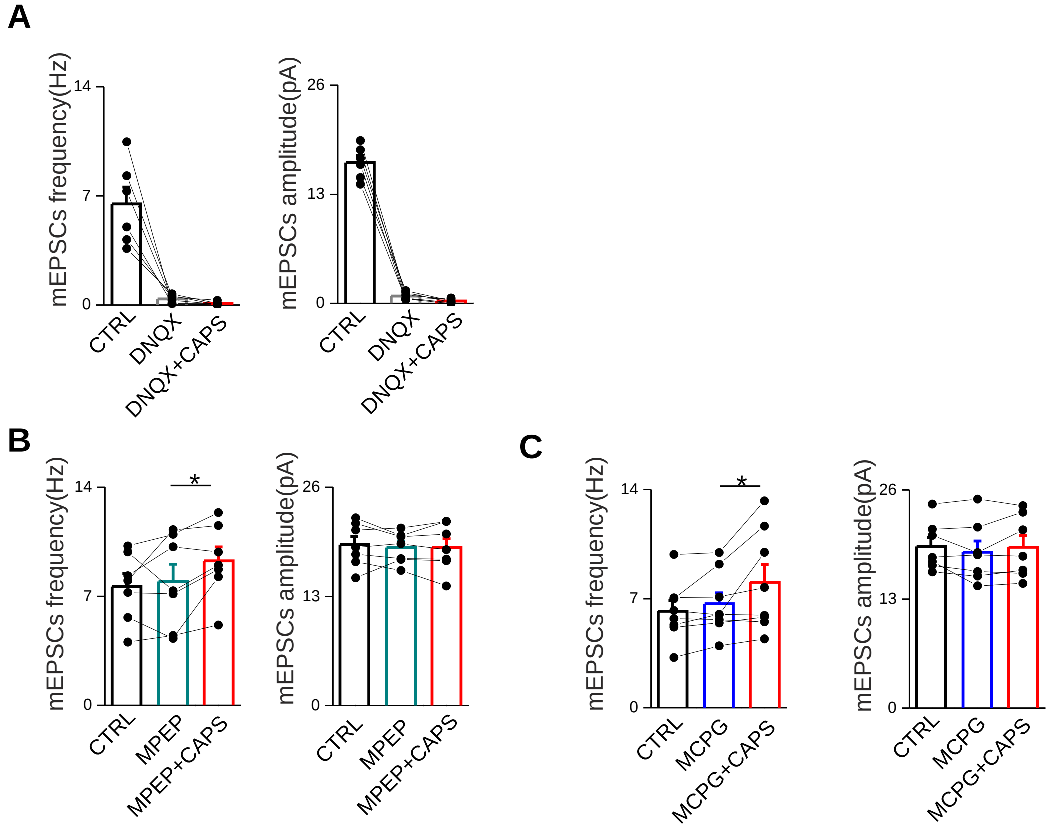

(**a**) The non-NMDA receptors antagonist, 6,7-dinitroquinoxaline-2,3-dione (DNQX, 20 µM) completely abolished both frequency and amplitude of mEPSCs (n= 6, paired T-test p= <0.001;). In these conditions, capsaicin increased neither frequency nor amplitude of mEPSCs, suggesting that downstream TRPV1 signalling is AMPA receptor mediated. The NMDA component was excluded in this type of experiments (resting membrane potential -70mV, [Mg2+] 2mM in the internal solution). (**b,c**) Glia-derived glutamate facilitates neurotransmitters release through presynaptic metabotropic receptors (Fiacco and McCarthy, 2004;Perea and Araque, 2007; Pascual et al. 2012); to investigate a possible involvement of these receptors in the presynaptic modulation of glutamatergic transmission upon TRPV1 activation, capsaicin was applied with the mGluR5 antagonist MPEP (50µM) or with the non-selective group I mGluR antagonist MCPG (100µM). In both assays capsaicin significantly increased mEPSC frequency (n=7, p= 0.040 paired sample T-test and n=8, p=0.042 paired sample T-test, respectively), suggesting that mGluR are not involved in the upstream TRPV1 signalling. Both MPEP and MCPG did not induce changes in basal synaptic properties. (Ctrl 7.62203 +/- 0.83 Hz, MPEP 7.95877+/- 1.10 Hz; Ctrl 19.16708 +/- 0.98 pA MPEP 18.83072 +/- 0.69 pA, n=7, p=0.61 and p=0.62 respectively, paired sample T-test; Ctrl 6.19 +/-0.67606 MCPG 6.66+/- 0.71Hz; Ctrl 19.46 +/-0.97 MCPG 18.66 +/-1.18,n=8, p=0.10 and p=0.13, paired sample T-test).

Supplementary Figure 13: Effects of ARN14988 and SB203580 on MV release and neurotransmission

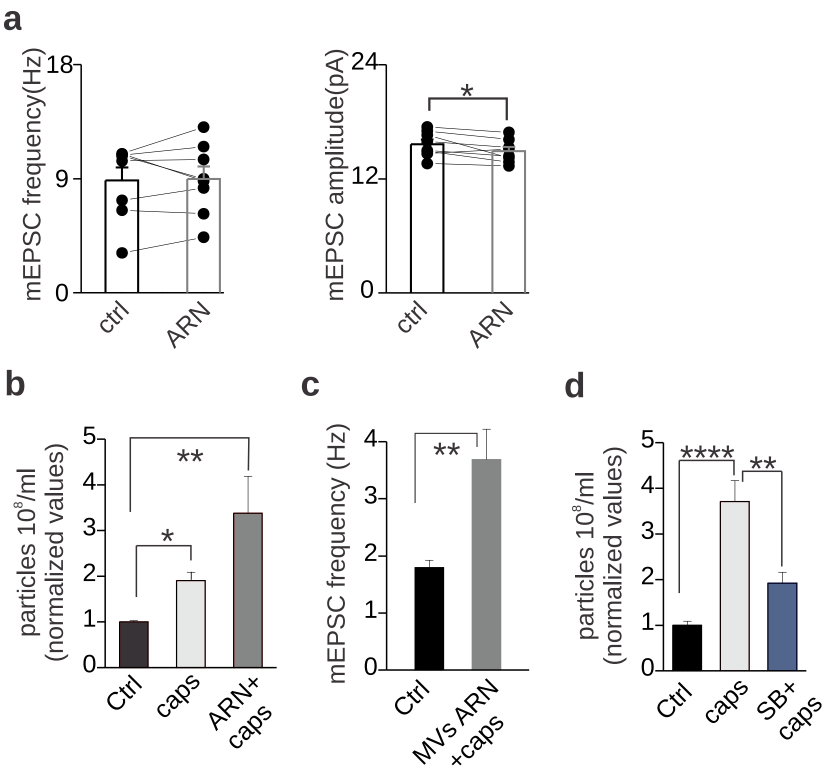

(**a**) Summary graph of mEPSC frequency (left) and amplitude (right) before (black empty bar) and after 3 µM ARN 14988 (gray empty bar) (n=10, p=0.84 and *p<0.05, Paired Sample t-test). (**b**) Microglia cell cultures were challenged with vehicle, caps or ARN+caps and MVs shedding were measured. Histogram shows the fold increase of total EV concentration detected by Nanosight upon stimulation of microglia cultures with vehicle (ctrl, black bar), capsaicin (light gray bar) or ARN+capsaicin (dark grey bar; One way Anova *p<0.05,**p<0.01 Dunn's Method for multiple comparison test). (**c**) Changes of mEPSCs evoked by MVs previously treated with ARN+caps (ctrl n=10, caps n=8, p<0.01 Mann-Whitney Rank Sum Test). (**d**) As in (b) but with 400nM SB203580 (blue bar; One way Anova ****p<0.0001 followed by Tukey’s multiple comparison test)

Supplementary Figure 14: Microglia morphology characterization

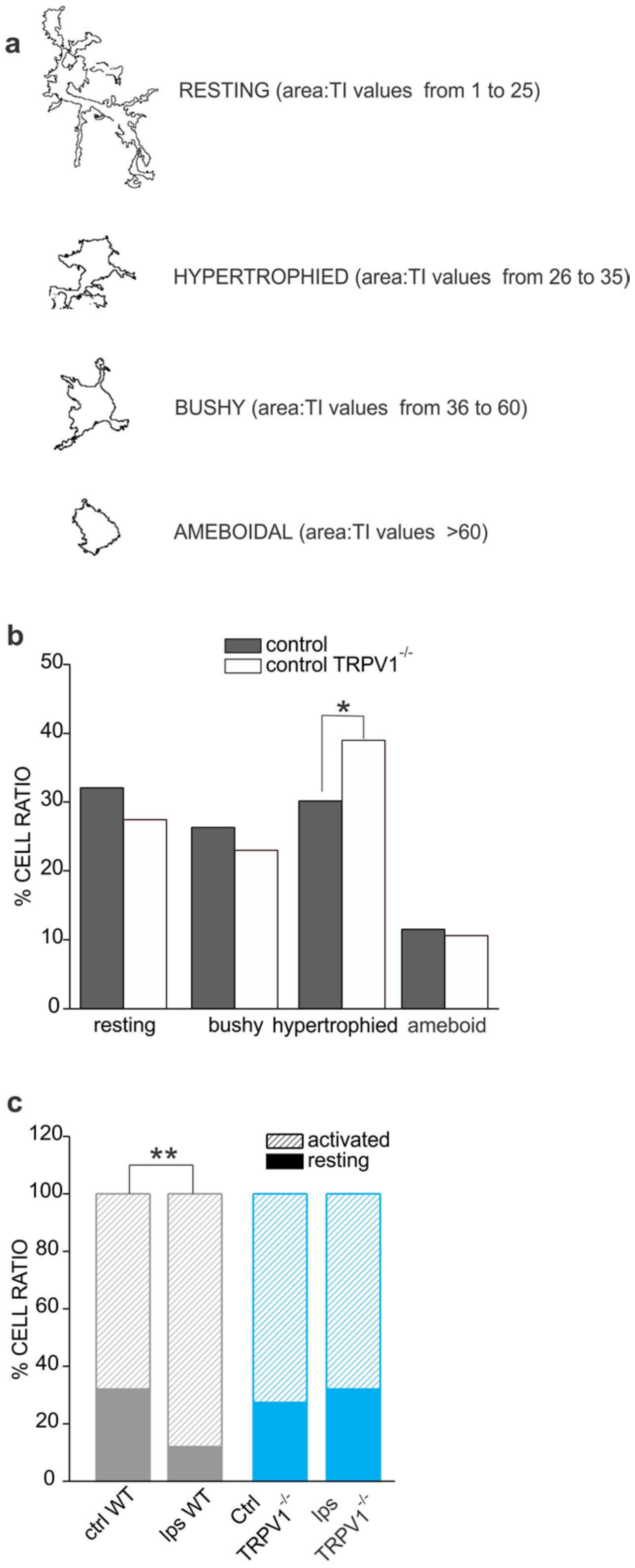

(**a**) Example of binary (digital) silhouettes of microglia cells from cortical slices acquired by transforming the tiff files of Iba1-immunoreactive cells to binary files by means of the ImageJ software. The cell area values and the TI were calculated for each silhouette and the obtained area/TI ratio value was used to classify each cell in a specific category (resting, ipertrophied, bushy and amoeboid).(**b**) Plot of stochastic sampling of microglia cells belong to a specific category from WT (black bars) and *TRPV1*$^{-/-}$ (empty bar) cortical slices (n=5 for both WT and *TRPV1*$^{-/-}$). Note that in no treated-*TRPV1*$^{-/-}$ tissue there is a higher amount of hypertrophied microglia cells than in WT. (**c**) Stack column graph showing a greater percentage of activated cells (striped bar) related to resting microglia (filled bar) in WT slices (grey bars) treated for 10 minutes with LPS (500ng/ml). The same treatment carried out on slices from *TRPV1*$^{-/-}$ mice (blue bars) produced no changes in the number of activated cells.
(*p<0.05, **p<0.01 Fisher's exact test).

Supplementary Figure 15: Electrophysiological features of CCI PNs in baseline conditions and after TRPV1 activation

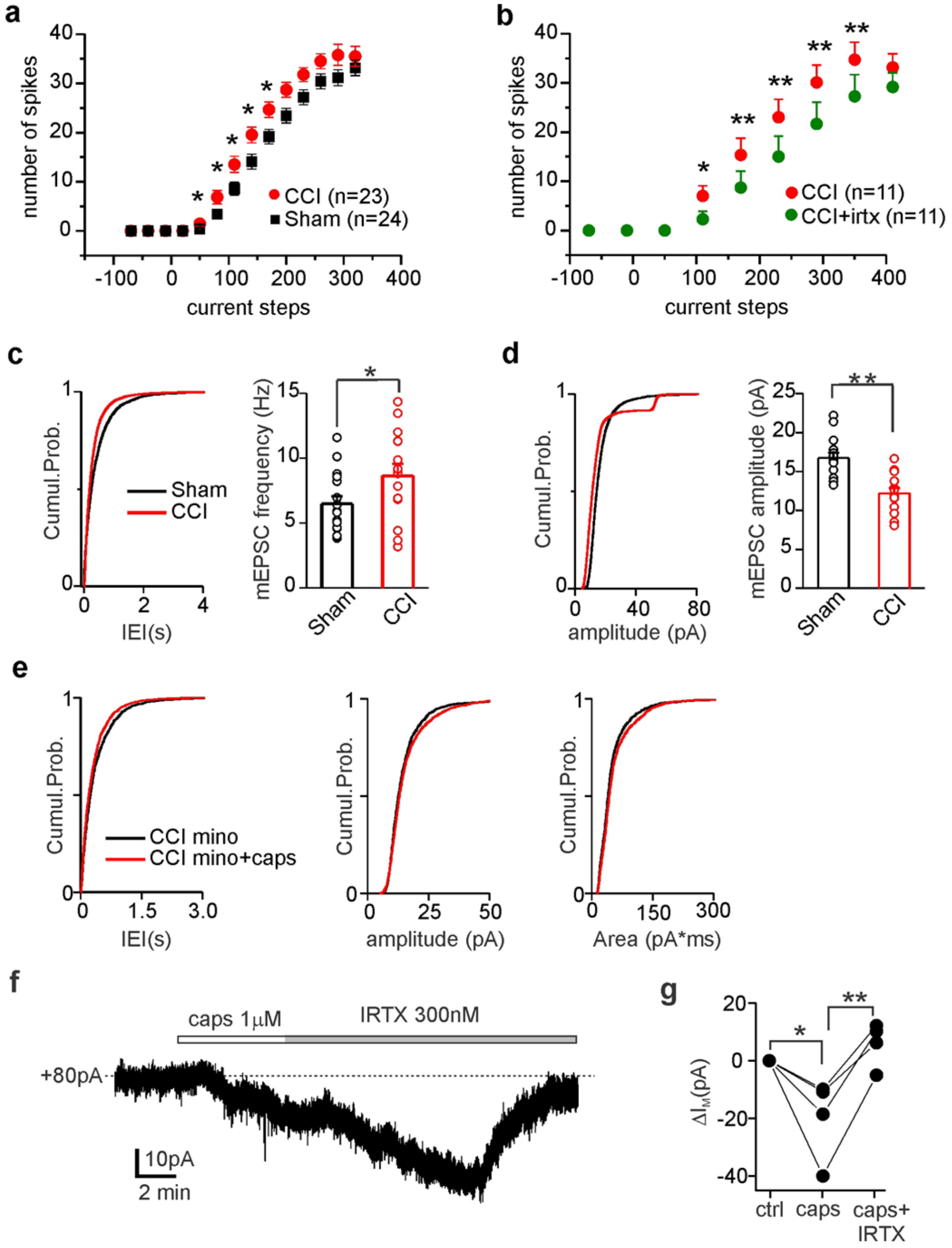

(**a**) Comparison between average input-out (input current/number of spikes) curves for the two conditions, shams (black dots) and CCI (red dots). The sciatic nerve ligation caused a leftward shift of the input current to action potential curves, with a significant increase of firing rate over a range of current injections. P<0.05 Mann-Whitney test. (**b**) Average input current/number of spike curves for control (red) and after IRTX (green) from CCI PN recordings. The inhibition of TRPV1s significantly reverted the leftward shift of the input-output curve induced by the surgery of the

sciatic nerve (*p<0.05, **p<0.005, Paired Sample Wilcoxon Signed Rank test). (**c**) On the left, cumulative probability curves of mEPSC interevent intervals for the total recorded neurons from sham (n=16, black line) and CCI mice (n=14, red line) (p<0.001 Kolmogorov-Smirnov test). Right, summary histograms and line series plots of mEPSC from PNs of shams and CCI mice (6.47±0.58 and 8.63±0.92 Hz shams [n=16] and CCI mice [n=14], respectively p<0.05 Two sample T-test). (**d**) Left, cumulative probability distributions of mEPSC amplitudes of total recorded PNs from shams (in black) and CCI (in red), p<0.001 KS test. Right, summary histograms and line series plots of mEPSC amplitudes from PNs of sham and CCI mice (16.76±0.65 and 12.2±0.67 pA, respectively, p<0.01 Two sample T-test. (**e**) Cumulative probability distributions of interevent intervals (left) amplitude (center) and area (left) of mEPSCs recorded from 4 cells of CCI mice in the presence of minocycline (p<0.01, KS test). (**f**) Voltage-clamp recording of layer 2/3 PN from a 4 week CCI mice in the presence of picrotoxin, APV, TTX, and DNQX. Application of capsaicin induced an inward current that was reverted by IRTX. (**g**) Summary plot of changes in I_M after application of capsaicin and subsequent application of IRTX (n=4, *p<0.05 **p<0.01).

Supplementary Figure 16: Development of allodynia after CCI.

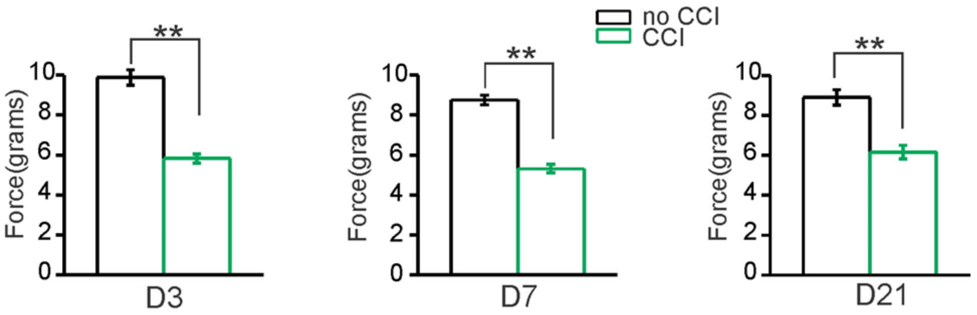

Mechanical threshold (force-grams) was measured in naive (no CCI, n=10) and CCI mice (n=11) at 3 (D3; $DF_{1,19}$; F 83,02; p<0.0001), 7 (D7; DF $_{1,19}$; F 118,19; p<0.0001) and 21 days (D21; $DF_{1,19}$; F 28,05; p< 0.0001) after neuropathy induction.

Supplementary Figure 17: Schematic summary of the proposed TRPV1 mechanism in the cortex of mice in physiological and neuropathic pain condition.

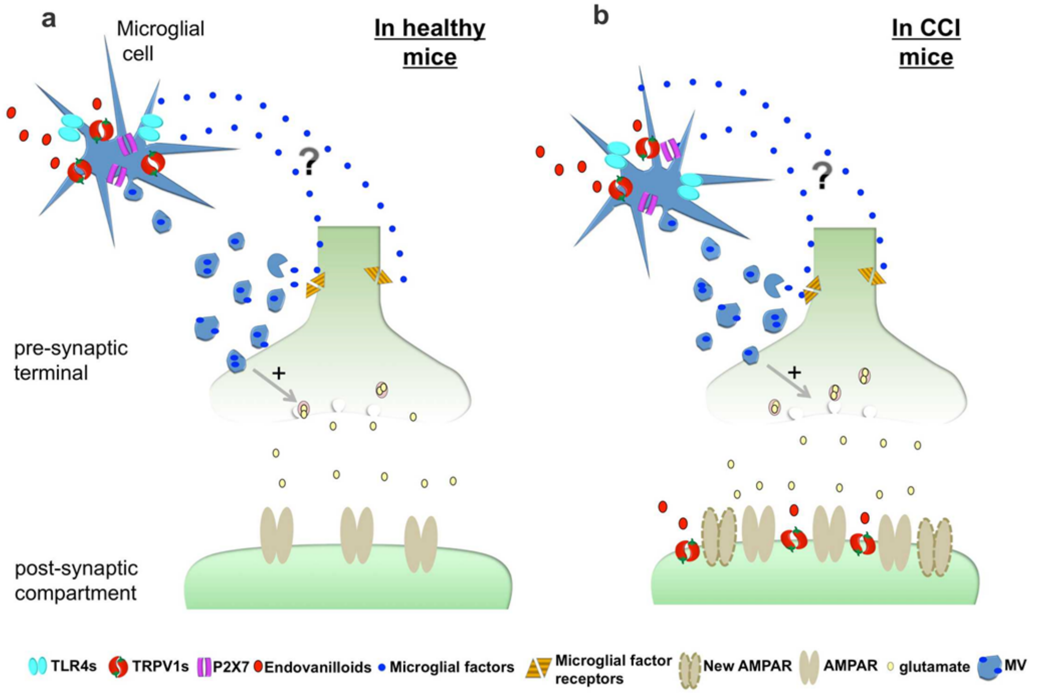

(**a**) In the cortex of healthy mice, TRPV1s are preferentially expressed in microglia. Activation of these channels induces the microvesicles (MVs) shed from microglia surface. Once released, these MVs may enhance spontaneous excitatory neurotransmission by increasing the ready releasable pool of vesicles together with the release probability. Additional players in the microglia to neuron communication could be microglial factors released either from the breakdown of MVs or directly from the microglia surface (such as IL-1β, NGF, ATP, NO, TNFα and glutamate). (**b**) In mice suffering from neuropathic pain, TRPV1 are also localized in principal cortical neurons, besides microglial cells. In this scenario, while microglial TRPV1 activation accounts for the increase of glutamate release, activation of postsynaptic TRPV1 directly affects pyramidal neuron excitability by changing membrane potential and likely increasing AMPA receptor trafficking.

Supplementary Figure 18.

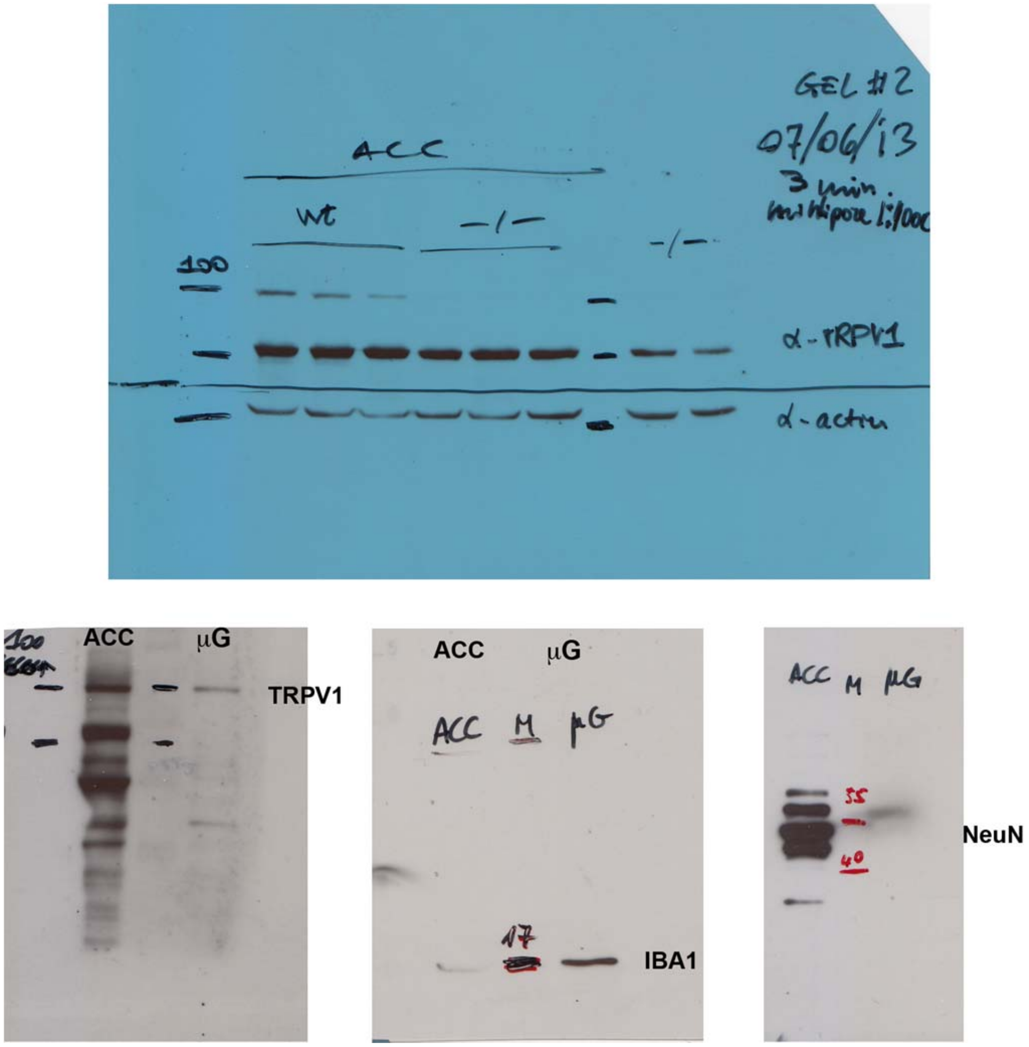

Full western blots from which the cropped images in Supplementary Figure 4.

SUPPLEMENTARY REFERENCES

1. Li, Y., Du, X. F., Liu, C. S., Wen, Z. L. & Du, J. L. Reciprocal regulation between resting microglial dynamics and neuronal activity in vivo. *Dev Cell* **23**, 1189-1202 (2012).

2. Ji, K., Akgul, G., Wollmuth, L. P. & Tsirka, S. E. Microglia actively regulate the number of functional synapses. *PLoS One* **8**, e56293 (2013).

3. Zhou, Q. et al. Decreased miR-199 augments visceral pain in patients with IBS through translational upregulation of TRPV1. *Gut* **65**, 797-805 (2016).

4. Jiang, G. et al. Expression levels of microRNA-199 and hypoxia-inducible factor-1 alpha in brain tissue of patients with intractable epilepsy. *Int J Neurosci* **126**, 326-334 (2016).

5. Luo, H., Cheng, J., Han, J. S. & Wan, Y. Change of vanilloid receptor 1 expression in dorsal root ganglion and spinal dorsal horn during inflammatory nociception induced by complete Freund's adjuvant in rats. *Neuroreport* **15**, 655-658 (2004).

6. McKelvey, R., Berta, T., Old, E., Ji, R. R. & Fitzgerald, M. Neuropathic pain is constitutively suppressed in early life by anti-inflammatory neuroimmune regulation. *J Neurosci* **35**, 457-466 (2015).

7. Latremoliere, A. & Woolf, C. J. Central sensitization: a generator of pain hypersensitivity by central neural plasticity. *J Pain* **10**, 895-926 (2009).

8. Barrientos, R. M. et al. Peripheral infection and aging interact to impair hippocampal memory consolidation. *Neurobiol Aging* **27**, 723-732 (2006).

9. Konsman, J. P., Parnet, P. & Dantzer, R. Cytokine-induced sickness behaviour: mechanisms and implications. *Trends Neurosci* **25**, 154-159 (2002).

10. Chen, T. et al. Postsynaptic potentiation of corticospinal projecting neurons in the anterior cingulate cortex after nerve injury. *Mol Pain* **10**, 33 (2014).

11. Zhuo, M. Cortical excitation and chronic pain. *Trends Neurosci* **31**, 199-207 (2008).

12. Bushnell, M. C., Ceko, M. & Low, L. A. Cognitive and emotional control of pain and its disruption in chronic pain. *Nat Rev Neurosci* **14**, 502-511 (2013).

13. Moreira, F. A., Aguiar, D. C., Terzian, A. L., Guimaraes, F. S. & Wotjak, C. T. Cannabinoid type 1 receptors and transient receptor potential vanilloid type 1 channels in fear and anxiety-two sides of one coin? *Neuroscience* **204**, 186-192 (2012).

14. Turkheimer, F. E. et al. The methodology of TSPO imaging with positron emission tomography. *Biochem Soc Trans* **43**, 586-592 (2015).

15. Marinelli, S. et al. Botulinum neurotoxin type A counteracts neuropathic pain and facilitates functional recovery after peripheral nerve injury in animal models. *Neuroscience* **171**, 316-328 (2010).

16. Fujita, H. et al. Effects of GM-CSF and ordinary supplements on the ramification of microglia in culture: a morphometrical study. *Glia* **18**, 269-281 (1996).

17. Szabo, M. & Gulya, K. Development of the microglial phenotype in culture. *Neuroscience* **241**, 280-295 (2013).

18. Chiurchiù, V. et al. Proresolving lipid mediators resolvin D1, resolvin D2, and maresin 1 are critical in modulating T cell responses. *Sci Transl Med* **8**, 353ra111 (2016).

19. Harris, J. A. et al. Anatomical characterization of Cre driver mice for neural circuit mapping and manipulation. *Front Neural Circuits* **8**, 76 (2014).

20. Ringkamp M, et al. Capsaicin responses in heat-sensitive and heat-insensitive A-fiber nociceptors. *J Neurosci*. **21**, 12 (2001).
